# Primer Binding Site (PBS) Profiling of Genetic Diversity of Natural Populations of Endemic Species *Allium ledebourianum* Schult.

**DOI:** 10.3390/biotech10040023

**Published:** 2021-10-13

**Authors:** Oxana Khapilina, Ainur Turzhanova, Alevtina Danilova, Asem Tumenbayeva, Vladislav Shevtsov, Yuri Kotukhov, Ruslan Kalendar

**Affiliations:** 1 National Center for Biotechnology, Korgalzhin Hwy 13/5, Nur-Sultan 010000, Kazakhstan; turzhanova-ainur@mail.ru (A.T.); asem.tumenbaeva2016@mail.ru (A.T.); xatabadich@gmail.com (V.S.); 2 Altai Botanical Garden, Yermakova Str 1, Ridder 070000, Kazakhstan; a-n-danilova@yandex.ru (A.D.); sumbembayev@gmail.com (Y.K.); 3 National Laboratory Astana, Nazarbayev University, Nur-Sultan 010000, Kazakhstan; 4 Helsinki Institute of Life Science HiLIFE, Biocenter 3, Viikinkaari 1, University of Helsinki, FI-00014 Helsinki, Finland

**Keywords:** *Allium ledebourianum* Schult., molecular marker, genetic diversity, iPBS amplification, DNA profiling

## Abstract

Endemic species are especially vulnerable to biodiversity loss caused by isolation or habitat specificity, small population size, and anthropogenic factors. Endemic species biodiversity analysis has a critically important global value for the development of conservation strategies. The rare onion *Allium ledebourianum* is a narrow-lined endemic species, with natural populations located in the extreme climatic conditions of the Kazakh Altai. *A. ledebourianum* populations are decreasing everywhere due to anthropogenic impact, and therefore, this species requires preservation and protection. Conservation of this rare species is associated with monitoring studies to investigate the genetic diversity of natural populations. Fundamental components of eukaryote genome include multiple classes of interspersed repeats. Various PCR-based DNA fingerprinting methods are used to detect chromosomal changes related to recombination processes of these interspersed elements. These methods are based on interspersed repeat sequences and are an effective approach for assessing the biological diversity of plants and their variability. We applied DNA profiling approaches based on conservative sequences of interspersed repeats to assess the genetic diversity of natural *A. ledebourianum* populations located in the territory of Kazakhstan Altai. The analysis of natural *A. ledebourianum* populations, carried out using the DNA profiling approach, allowed the effective differentiation of the populations and assessment of their genetic diversity. We used conservative sequences of tRNA primer binding sites (PBS) of the long-terminal repeat (LTR) retrotransposons as PCR primers. Amplification using the three most effective PBS primers generated 628 PCR amplicons, with an average of 209 amplicons. The average polymorphism level varied from 34% to 40% for all studied samples. Resolution analysis of the PBS primers showed all of them to have high or medium polymorphism levels, which varied from 0.763 to 0.965. Results of the molecular analysis of variance showed that the general biodiversity of *A. ledebourianum* populations is due to interpopulation (67%) and intrapopulation (33%) differences. The revealed genetic diversity was higher in the most distant population of *A. ledebourianum* LD64, located on the Sarymsakty ridge of Southern Altai. This is the first genetic diversity study of the endemic species *A. ledebourianum* using DNA profiling approaches. This work allowed us to collect new genetic data on the structure of *A. ledebourianum* populations in the Altai for subsequent development of preservation strategies to enhance the reproduction of this relict species. The results will be useful for the conservation and exploitation of this species, serving as the basis for further studies of its evolution and ecology.

## 1. Introduction

Endemic plants constitute an integral part of flora and fauna, and the extinction of these species can lead to significant ecological changes. Anthropogenic transformation of habitats is the greatest threat to the ecological niches of rare and endemic plant species. This leads to the isolation and disappearance of endemic populations, which significantly changes the ecological structures of local flora and fauna. The number of weed species that replace balanced and stable populations increase concurrently, and consist of a large number of species and their forms [1]. The negative impacts of anthropogenic factors (arable land expansion, deforestation, uncontrolled collection of rare and endemic plant species, overgrazing of animals, and the development of industrial production) along with global climate change are the root causes behind the loss of botanical diversity in natural ecosystems. The increasing detrimental impact of human activities occurs precisely in areas with significant species diversity and endemism, and in this regard, the study of plant community biodiversity is an urgent problem for many countries [2]. The biodiversity conservation of endemic species is of great fundamental importance for the health of global ecosystems, as it is these species that face a significantly higher risk of extinction and require additional conservation efforts [3,4]. The unique mountain system of Altai, known all over the world for its exceptional natural wealth of flora and fauna, is one of the global centers for plant biodiversity and is considered one of the loci of vascular plant endemism, included in the “Global 200” list [5]. The flora of the Kazakhstan Altai is considered one of the richest, as it contains more than 40% of the vascular plant species composition of Kazakhstan, which indicates that this region is one of the focal points for the formation of Asian flora. The vegetation of the Altai Mountains is composed mainly of typical Siberian flora, which are not represented anywhere else in Central Asia. The changing mountain, steppe, and desert landscapes, formed over a long geological history, which form a variety of ecological and soil-climatic conditions, create a high level of local endemism of the existing flora. The *Alliaceae* families compose nearly 30% of the area’s endemics [6,7]. Despite that N.I. Vavilov named Central Asia as the origin of cultivated *Allium* species, only approximately 20% out of 125 species have been partially studied, including ca. 30 endemics growing in the territory of Kazakhstan [8]. The genetic diversity of rare and endemic *Allium* species growing in the Kazakhstan Altai territory have not been studied sufficiently, and certain species require protection measures. One of these narrow endemic species, found only in the Altai Mountains of Russia, Mongolia, China, and Kazakhstan, is a poorly studied relic of *Allium ledebourianum* Schult. Its narrow distribution area along with the influence of anthropogenic factors significantly reduces the number of populations of this wild species. The increasing detrimental impact of human activities is highly damaging to such endemic and poorly studied species [9,10]. Uncontrolled green mass harvesting, mowing, and livestock grazing led to the thinning of thickets, a reduction in habitat, and a violation of the population age composition, especially near settlements. This species is of scientific interest primarily as a narrow endemic species in need of emergency protective actions. Since 1998, the species has been included in the Red Data Book of the Altai Territory [11]. Like many endemic species, *A. ledebourianum* exists as small, isolated populations, which are characterized by a decrease in the level of genetic variability due to the negative consequences of genetic drift. The low level of genetic polymorphism typical of endemic species can be caused by the narrowness of the distribution range and the small number of populations [12]. Endemic species, represented by small, fragmented populations, especially those located in isolated mountain systems, require careful research, as the study of their genetic diversity and population structure is important for conservation and appropriate genetic management. Some populations are sporadic and are sometimes represented by several specimens. A conservation and reproduction strategy is a very urgent task for preserving the biodiversity of the natural flora of Kazakhstan and should be addressed using modern approaches. In population and genetic studies, when choosing the optimal type of molecular markers, it is necessary to consider not only the features of the technology used, but also the uniqueness of the studied species. The most accessible molecular marker systems are ineffective and uninformative for poorly studied endemic species [13,14,15]. Contemporary molecular genetic analysis methods provide more precise and objective data on the genetic structure of populations [8,16,17]. Molecular genetic methods based on the polymorphisms of certain genome sequences or proteins are used to investigate the genetic polymorphism of plant species. Various PCR-based DNA fingerprinting methods, such as Random Amplified Polymorphic DNA (RAPD) [18], Inter Simple Sequence Repeat (ISSR) [19], and Amplified Fragment Length Polymorphism (AFLP) [20], have been used to study various *Allium* species. These PCR-based molecular markers are quite effective for determining genetic diversity, but each method has limitations in terms of reproducibility, cost, or development method. Molecular microsatellite markers or SSRs (Single Sequence Repeats) would be a promising alternative if such markers were developed for all onion species. However, these PCR-based molecular markers are expensive at the initial stage of development and require the comparative genome sequencing of several genetically distant genotypes for each species. Subsequent comparative bioinformatics analyses of these genomes are also required to identify polymorphic SSR loci and select efficient primer pairs for PCR. Such work is complex and expensive and is usually only required for critical crops that require such markers for identification and genotyping of the breeding lines. To investigate intraspecific genetic polymorphisms of *Allium* wildlife relict species, using markers characterized by wide genome distribution would be ideal and, above all, markers accessible for any species, including those that have not been studied. Such DNA genetic markers include all PCR-based DNA fingerprinting method variants of the RAPD method [18], such as ISSR [19], including a new method, Palindromic sequence-targeted (PST) PCR [21,22] and other approaches [23]. This list can also be supplemented with methods based on interspersed repeat sequences in the genomes, including a range of transposable elements, 5 S rRNA- and tRNA-related sequences, regions of certain promoters, and introns. These include PCR markers based on conserved sequences for various retrotransposon classes [24,25]. For some plant species, 45–90% of the genome consists of long-terminal repeat (LTR) retrotransposon, mobile genetic elements, the number of which changes dynamically during the evolution of the species and its adaptation to the environment [26,27]. These element types share a common unique feature: all classes of mobile genetic elements and their sequences contain short but conserved sequences among numerous classes of mobile genetic (transposons, retrotransposons) or related elements. These sequences can be used universally for plants and animals and for species under study for the first time. Eukaryotic genomes consist of significant amounts of retrotransposons, including LTR retrotransposons and non-LTR retrotransposons. Despite its gigantic size, the *Allium* species genome is also mostly represented by repeating sequences of LTR retrotransposons, satellite DNA, tandem repeats, and DNA transposons; more than 90% of the onion genome consists of repeated elements. Analysis of the sequences genomic composition of the repeatome, carried out on several species of *Allium* using Next Generation Sequencing (NGS), revealed that Gypsy and Copia elements were most common for the LTR retrotransposon classes [28,29,30]. Analysis methods and methods for studying interspersed repeat sequences in genomes, represented, for example, by the LTR retrotransposon classes, can be used to study the biodiversity of various *Allium* species. This applies not only to cultivated species but also to wild species [31,32]. The genetic polymorphism research method in plant species using retrotransposons is as simple and accessible as RAPD [18]. LTR retrotransposons are the most abundant component of the eukaryotic genome. Due to the large copy number of LTR retrotransposons and their abundance in eukaryotic genomes, the use of conserved sequences of elements as PCR-based DNA fingerprinting is convenient as an effective method. When compared with other markers, DNA fingerprinting methods based on LTR retrotransposons can be used to assess genetic polymorphism and evolutionary and phylogenetic studies [23,33]. Variations of these markers have been used in humans and in various species of fungi, plants, and animals [2,10,31,34,35]. Many mobile genetic elements “mix” with each other during inter-and intrachromosomal recombination, which brings highly conserved sequences closer together and makes PCR amplification possible [36,37]. These highly conserved sequences for retrotransposons include the tRNA priming binding site (PBS) when initializing LTR retrotransposon replication through RNA reverse transcription and integration of resultant cDNA into another locus. Sequences of the PBS region are complementary to at least 12 nucleotides of the tRNA sequences, which is already sufficient for use as PCR primers. As retrotransposon sequences are frequently near each other in inverted orientation, PBS sequences are accessible when used for DNA amplification for most eukaryotic species with large genomes, such as plants, fungi, animals, and humans. PCR primers complementary to short, interspersed repeats enable the amplification of the region between these repeats if the distance between the repeats does not exceed the processivity of the DNA polymerase used. Therefore, PCR methods for interspersed repeats will produce longer PCR fragments if the repeat sequences are rare in the genome and short PCR fragments if the repeats are frequent. This approach, based on PCR between overlapping mobile genetic elements, can thus easily be adapted to any eukaryotic species for the rapid detection of molecular genetic polymorphisms, even in an agarose gel. Thus, the use of PCR methods based on highly conserved sequences of interspersed mobile genetic elements allows efficient and versatile detection of polymorphisms for nearly all eukaryotic species [38]. Such markers were used to assess the genetic polymorphism in rare and poorly studied plant species [14,39,40,41]. However, insufficient data are available on the use of such markers for studying the biological diversity of endemic onion species living in stressful conditions, although retrotransposons are involved in plant adaptation to stressful environmental conditions. Under normal conditions, retrotransposons are at rest, but various forms of stress can cause their transcription and activation. Stress-induced activation of retrotransposons has been described for many plants; it occurs when exposed to salt or cold/heat stress, or when exposed to pathogenic microorganisms [42,43,44,45]. The stress response mechanism in plants of the *Allium* family is not well studied; in total, more than 400 genes are known that are expressed in response to stress [46]. The insertion of stress-activated retrotransposons into the coding regions of a gene can lead to a change in the expression level of these genes or may affect the transcription of neighboring ones [35]. Therefore, bursts of activity of retrotransposons and transposons can be genetically fixed under stressful conditions, especially for plants propagating vegetatively. The induced genetic rearrangements and insertions of mobile elements in the areas of active euchromatin contribute to changes in the genome, leading to “genome stress” [43,47,48]. Transcriptionally active retrotransposons can remodel gene structure and rewire gene networks in a relatively short evolutionary time frame, as their activity is induced by stressful environmental conditions [49]. The prolonged and multiple chromosomal changes occurring under the influence of active mobile elements can potentially increase the adaptive potential of individuals to stressful conditions [50,51]. In this regard, the use of highly conserved sequences for retrotransposons including tRNA PBS as markers in assessing the biodiversity of the narrowly localized endemic *A. ledebourianum* is novel and relevant. Ours study is devoted to examining the genetic diversity of the endemic species *A. ledebourianum* using iPBS (inter-Priming Binding Site) amplification DNA profiling markers. The genetic diversity analysis of *A. ledebourianum* populations in the territory of Kazakhstan Altai was carried out for the first time, enabling the clarification of the genetic structure of this narrow endemic species’ populations. Our research results are relevant in terms of developing a strategy for the conservation of natural genetic diversity and re-introduction of endemic species.

## 2. Materials and Methods

### 2.1. Plant Material

**Figure 1:**
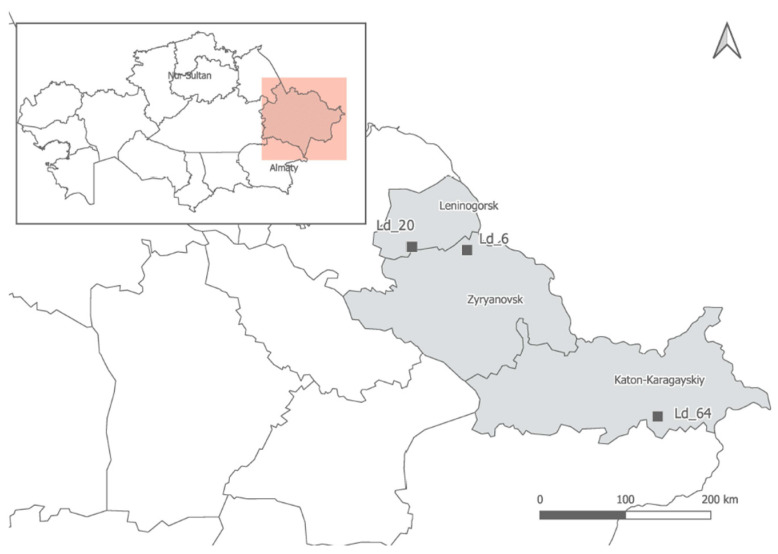
Locations of sample collection of *A. ledebourianum* in the Kazakhstan Altai territory.

Samples of three Kazakhstani populations of *Allium ledebourianum*, collected in places of their natural growth in the Kazakhstan Altai territory, were used as study objects. Expeditions to collect samples were carried out after snowmelt, guided by weather conditions and temperature conditions. At least 10 plants were selected for each population. Populations were distant from each other and were growing relatively evenly over the entire occurrence area. For each population, the coordinates (latitude and longitude) and the absolute altitude of its location were determined using a Garmin GPS 72 H GPS navigator (Olathe, Kansas, United States). The locations for the populations are shown in Figure 1. The plants of each population were numbered in chronological order. The plant material was placed in plastic bags, stored on ice under expedition conditions for transportation, and then at −35 °C in the laboratory.

The geobotanical descriptions of communities with *A. ledebourianum* were carried out on the test plots, each of which was 250 m^2^ in size. Ontogenetic age states of the plants and the spatial structure of *A. ledebourianum* populations were determined using standard centenopopulation methods [52,53,54].

*Allium ledebourianum* populations were assessed based on quantitative morphological characters, which were taken into account only in plants of a mature ontogenetic state. Generative age states were identified according to the recommendations of NI Fedorov [55]. Samples containing up to 30 samples were used for population analysis (due to the small number of certain *A. ledebourianum* populations).

### 2.2. DNA Isolation and PCR Amplification

Total plant DNA was extracted from fresh plant leaves (50–100 mg) using a modified acidic-CTAB extraction buffer (1.5%, 2 M NaCl, 10 mM Na_3_EDTA, 100 mM HEPES, pH 5.3) in the presence of RNAse A [56]. After DNA precipitation with 2-propanol and intensive washing of the pellets with 70% ethanol, the DNA pellets were dissolved with 1× TE buffer (1 mM EDTA, 10 mM Tris-HCl, pH 8.0). The DNA quality was checked spectrophotometrically with a Nanodrop apparatus (Thermo Fisher Scientific Inc., Waltham, MA, USA) and with 1% agarose gel.

PBS primers (Table 1) were used to assess the genetic diversity of *A. ledebourianum* populations [57].

The genetic variability of endemic *A. ledebourianum* samples was analyzed using PBS primers designed by Kalendar et al. [57]. PCR reactions were performed in a 25-µL reaction mixture. Each reaction mixture contained 25 ng of template DNA, 1× Phire^®^ Hot Start II PCR buffer with 1.5 mM MgCl_2_, 1 µM primer, 0.2 mM each dNTP, and 0.2 µL Phire^®^ Hot Start II DNA polymerase (Thermo Fisher Scientific Inc., Waltham, MA, USA). PCR amplification was carried out in a SimpliAmp ™. Thermal Cycler (Thermo Fisher Scientific Inc., Waltham, MA, USA) under the following conditions: an initial denaturation step at 98 °C for 1 min, followed by 30 amplifications at 98 °C for 5 s, at 50–57 °C (depending on primer sequence) for 20 s, and at 72 °C for 60 s, followed by a final extension of 72 °C for 3 min.

All PBS primers were tested to assess the genetic diversity of *A. ledebourianum* for DNA profiling. There were no limitations in the number of PBS primers used in the previously published study [57]. However, for this study, we wanted to only choose primers that were convenient to analyze and that generated enough clear bands that could be monitored in all the samples. Therefore, we used certain “comfortable” PBS primers for our work. More PBS primers could be included, but adding additional primers no longer added new information, as the number of polymorphic bands obtained was more than enough for this work. PCR products were separated by electrophoresis at 70 V for 12 h in 1.2% agarose gel with a 1 x TBE buffer. A Thermo Scientific (100–10,000 base pairs) GeneRuler DNA Ladder Mix (#SM0332) was used as a standard DNA ladder. The PCR products were visualized with a PharosFX Plus Imaging System (Bio-Rad Laboratories Inc., Hercules, CA, USA) with a resolution of 50 µm, after staining with ethidium bromide. PBS primers generated in the PCR yielded clearly distinct amplification products, showing considerable variability among *A. ledebourianum* plants from different populations.

### 2.3. Data Scoring and Analysis

To study the genetic diversity of *A. ledebourianum*, we only used clear bands that can be followed for all the samples. Even if the bands were deformed during electrophoresis, we monitored these bands for correspondence to a specific band in the other samples. Common bands, or bands characteristic of most of the samples, were an excellent source for tracking and controlling all the bands. Bands above 2 kb were difficult to separate by electrophoresis, and we therefore did not analyze them. Analyzing the short bands was most straightforward, as they were easily followed for all samples. A band of unique size corresponds to a unique locus, and heterozygotes on the band were not considered. To construct the binary matrix, the PCR fragments were scored as present (1) or absent (0). The GenAlex 6.5 program (operates within Microsoft Excel) [59] was used to calculate the total number of alleles, the Shannon information index (I), the index of genetic differentiation (PhiPT) between populations, and the number of private alleles in the population. The analysis of molecular variance (AMOVA) between populations and within populations was also calculated using GenAlex 6.5. A dendrogram was constructed using the UPGMA method [60]. Correlation analysis of genetic variation and species productivity parameters using Spearman rank correlation coefficient was performed in the R environment [61].

### 2.4. Allium ledebourianum Sample Collection and Characteristics

The species *A. ledebourianum* (2n = 16) is a unique endemic, whose populations are found only on the Altai ridges (Table 2). The plants have a long cycle (35–50 years) of ontogenetic development, resulting in gradual accumulation of vegetative and generative individuals in the populations. The vegetation period only lasts 5–6 months, from April (when the shoots grow under snow) until September (when the ripe seeds crumble next to the completely dried mother plant). The species is a hygrophyte, i.e., the root system is located in the surface layer of the soil, and any decrease in the water level is a stress factor. Prolonged stagnation of water prevents the normal growth of young plants because cold weather leads to the formation of ice crust, resulting in the mass death of seedlings [61].

**Figure 2:**
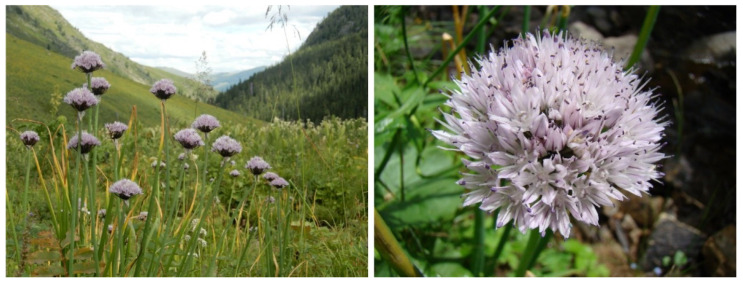
Plants of *A. ledebourianum* (flowering 14 July 2020, Southern Altai) (photo by I. Satekov).

We only studied *A. ledebourianum* populations unaffected by anthropogenic activity that were found on the Ivanovsky, Lineisky (Western Altai), and Sarymsakty (South Altai) ridges 1100–1900 m above sea level. Despite the significant spatial isolation of the populations, the species grows in approximately the same ecological conditions: all identified populations of *A. ledebourianum* have a narrow ecological confinement to places of increased moisture. The populations were located in places that allow a significant amount of moisture to accumulate during the entire growing season, e.g., mountain riverbanks or swampy meadows on organic-rich moist chernozem soils (Figure 1). The species is characterized by elongated or cylindrical bulbs, up to 0.5–1.0 cm thick, with grayish-brown shells. The plants have 40–80 cm tall stems, tubular leaves, and the inflorescence is a spherical umbrella with pink-purple flowers (Figure 2).

The ecological conditions of the *A. ledebourianum* populations in the Kazakhstan Altai territory are presented in Table 3.

**Figure 3:**
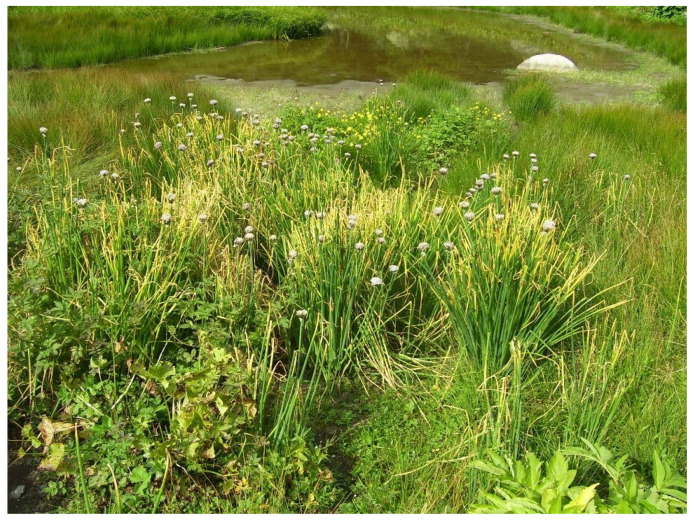
Plants of *A. ledebourianum* on the ridges of Western Altai (August 2020) (photo by N. Premina).

Population LD20 is located in the Western Altai territory at an altitude of 1170 m above sea level, in relief depressions where conditions are created for the accumulation of sufficient thawed moisture. Population LD20 is below sea level relative to the other two populations. Onion plants are located mainly diffusely, along stream banks and temporary gutters, in sunny and well-ventilated spaces. Well-moistened and humus-rich soil creates good conditions for plant growth, and large bushes are formed (Figure 3). However, the species does not withstand competition from meso- and xerophytic meadow vegetation at later stages and falls out of the phytocenosis. One- to two-year-old seedlings were recorded only in the coastal zone.

The vegetation that forms this phytocenosis is represented by species that are resistant to soil moisture. The above-soil layer is loose, formed mainly of leaf litter, which decomposes more slowly due to water stagnation during the spring–autumn periods, and high grass that does not allow sunlight to pass through. We assigned this population as a full member, i.e., a normal type where all age categories are represented. Single plants affected by rust, gray mold, and powdery mildew were identified, but this does not significantly affect the population size and structure. The other two populations (LD64 and LD6) are located at the same altitude of 1925 m above sea level. LD6 is in Western Altai, while population LD64 is located to the south, in the South Altai territory. However, differences in soil and climatic conditions affect the species composition of phytocenoses and, accordingly, the structure of *A. ledebourianum* populations. The LD6 population is under conditions of excessive moisture, and the plants are scattered, mainly on hummocks. This is largely because the soil freezes to a depth of 35 cm prior to snowfall. Snow cover height can reach 150 cm during some years, and snowmelt therefore occurs only in April, but the frozen soil contributes to long stagnation of the melt water (until the end of June). Damage by fungal diseases was noted due to excess moisture. The aging population is mainly represented by generative individuals, and the proportion of young 1–2-year-old seedlings is insignificant, possibly due to seeds and young seedlings being washed out by melt water or dying due to deep soil freezing and ice crust formation. The LD64 population has the densest spatial structure. The species composition of the phytocenosis is very poor, which allows *A. ledebourianum* plants to form dense clumps consisting of several generative stems in places of excessive moisture. Humus-rich soil substrate and the presence of a well-developed above-ground cover creates favorable conditions for *A. ledebourianum* growth and development. Despite the high substrate moisture content, no fungal diseases were detected in the LD64 plants, as the habitats are open and well warmed by the sun. The population is full-lived, and the dominant position is occupied by vegetative and generative individuals, which indicates rather favorable conditions for the population’s existence. The characteristics of the *A. ledebourianum* samples, carried out on 20 measurements, are presented in Table 2. As *A. ledebourianum* predominantly reproduces via seeds, we mainly considered the characteristics of the generative organs. The results showed that the populations differ in almost all studied morphological characteristics. The maximum value of the generative shoot height varied from 108 cm in the LD20 population plants to 32 cm in the LD6 population plants. The maximum number of bolls per inflorescence (995 pieces) was also noted in the LD20 population, the minimum (565 pieces) in plants from the LD64 population. Our results show that the number of inflorescences, fruits (capsules), and seeds decreases, and so does the weight of 1000 seeds with an increase in habitation altitude in *A. ledebourianum* plants. Plants from the low-mountain population LD20, located in relatively favorable conditions, where optimal conditions are formed that contribute to an increase in organic components in the soil, were found to have plant habitats at higher altitudes. The average height of the generative shoot was also higher. We observed a significant decrease in these studied traits in plants from the other two high-altitude populations (1925 m above sea level). We noted the absence of formed bulbs in the plants of all populations. For the rest of the characteristics, we did not observe any regularities in the decrease in productivity indicators due to an increase in the height of the range.

## 3. Results

### 3.1. iPBS Loci Variability Polymorphism Analysis of Natural A. ledebourianum Populations

**Figure 4:**
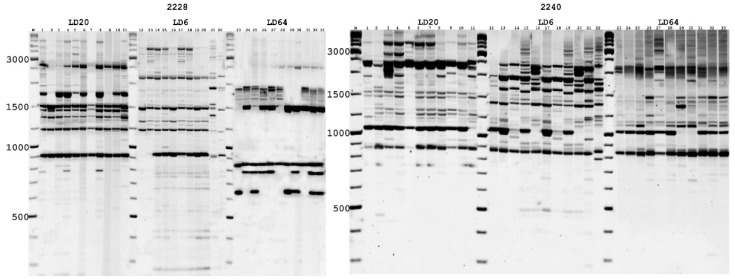
Electrophoretic pattern of *A. ledebourianum* fragments using PBS primer 2228 and 2240. 1. M—Thermo Scientific GeneRuler DNA Ladder Mix (100–10,000 bp). Samples of LD20 (1–10), LD6 (11–21), and LD64 (22–33).

To assess the genetic polymorphism of natural *A. ledebourianum* populations, the most informative PBS primers (2228, 2240, and 2395) were selected out of 22 alternatives, and they were preliminarily analyzed in accordance with Kalendar et al. [57]. The ability to generate clear and reproducible PCR amplicons was the criterion used for primer selection. Electrophoretic separation of PCR products yielded amplicons with sizes ranging from 200 to 6000 bp. The number of fragments amplified, and their distribution profile depended on each specific population and on the primers used (Figure 4).

Electropherograms of the amplification results demonstrate the presence of both common amplicons that are typical for samples of all populations, and unique ones that are typical for each population. To confirm the reproducibility of the analysis results, we carried out the studies with each DNA sample at least three times. This made it possible to identify reproducible specific iPBS profiles that are unique for each *A. ledebourianum* accession. Amplification profile data obtained using PBS primers was assessed with the DNA profiling method using informative parameters such as amplicon polymorphism (%) and polymorphic information content (PIC). These indicators reflect the primer resolutions for detecting differences between *A. ledebourianum* genotypes (both within and between populations). All data on DNA fingerprinting analysis are given in Table 3. The primers used in this study generated many fragments, which totaled 628 in number. The minimum number of amplified fragments was obtained using primer 2395, the maximum (258) using primer 2228. Depending on the primer, the proportion of polymorphic bands varied from 38% to 49%, while the average level of polymorphism was 42%. The polymorphism index (PIC) had the minimum value (0.965) when using primer 2240, the minimum when using primer 2228. In general, all the PBS primers used in our study have high resolution for identifying genetic polymorphism in natural *A. ledebourianum* populations.

### 3.2. Analysis of the Genetic Differences between A. ledebourianum Populations Based on iPBS Amplification

**Figure 5:**
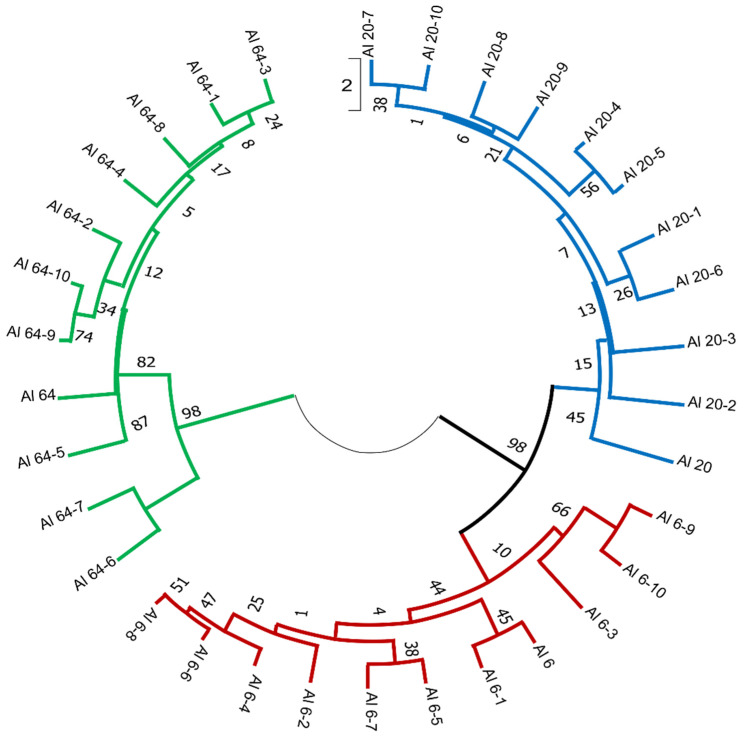
An UPGMA dendrogram analysis of 3 *A. ledebourianum* populations.

PCR amplification profiles obtained through the iPBS amplification were used to estimate each population’s genetic diversity. Additionally, the molecular profile of each *A. ledebourianum* population was examined individually to study intrapopulation diversity. The main indicators of genetic variability were determined based on the iPBS profiles in each *A. ledebourianum* population (Table S1). The populations were ranked according to the number of amplified fragments, where the LD6 population had the maximum number (251 fragments) and the LD64 population had the minimum (156 fragments). The minimum number of polymorphic bands was concurrently observed in the LD6 population, while this indicator was the same in the other two, i.e., at the level of 40%. Ranking by the Shannon diversity index in descending order was: population LD64 (0.152) > population LD6 (0.131) > population LD20 (0.108), with a mean of 0.195. The similarity of the Nei gene diversity between the *A. ledebourianum* populations ranged from 0.108 to 0.152. Results of the molecular variance (AMOVA) demonstrated that most (67%) of the molecular variation in *A. ledebourianum* populations exists among individual populations and within populations (33%), respectively. Permutation tests (based on 999 permutations) suggest that the overall Φ PT was significant (Φ PT = 0.674, *p* < 0.001; Table S2), which indicates that differences among populations are significant. The revealed differentiation of *A. ledebourianum* populations, due to the presence of unique genotypes, made it possible to carry out clustering, reflecting their genetic diversity. Cluster analysis and dendrogram construction were carried out using the UPGMA method (Unweighted Pair Group Method with Arithmetic mean). The results of the cluster analysis showed that the results of the iPBS amplification profiling positively correlated with the geographic data of the collection sites and with the morphological analysis of the *A. ledebourianum* samples. Three clades were identified as a result of cluster analysis based on iPBS profiling data (Figure 5), and two populations (LD6 and LD20) formed a single clade. This is because these populations are also the closest to each other geographically and according to the results of our morphological and iPBS profiling analyses. The LD64 population, located in the Southern Altai territory, differs significantly from the other studied populations based on its iPBS profiling, and is resultingly distinguished into a separate clade on the dendrogram.

### 3.3. Relationship between the Analyzed Parameters of A. ledebourianum

We assumed that the degree of intrapopulation diversity of specific *A. ledebourianum* populations is determined by habitat conditions. For example, the populations’ heights above sea level correlate positively, taking into account Spearman’s rank correlation coefficient, with the phenotypic variability of the studied samples, and are shown in Table S3. The height of the generative shoot correlated positively with seed productivity. Taller shoots receive more sunlight, do not experience competition from other plants, and therefore produce many more fruits and seeds. Correlation analysis shows that a negative correlation was found between the population heights and the seed productivity indicators (actual and potential seed production, and the seed production coefficient). High and medium correlation were revealed only by the number of formed bolls in inflorescences; however, the coefficient of reliability does not allow us to judge that this dependence is not random. It is possible to reliably consider the influence of the number of flowers in the inflorescence on the potential and actual seed production, along with the dependence of the actual seed production on the potential one. We additionally observed a high degree of reliability of the negative correlation between the formation of bolls per plant and soil germination of *A. ledebourianum*. Finally, we analyzed the relationship between genetic diversity indicators, loci polymorphism, and some phenotypic characteristics of *A. ledebourianum* plants (Table S4). We found a significant positive correlation between genetic diversity indicators and plant seed productivity. With an increase in seed numbers, the indices of genetic diversity also increase, along with the heterogeneity of the population.

## 4. Discussion

The narrowing of the genetic diversity of species leads to a decrease in plasticity, creating the risk of species abundance decreasing under the negative impact of environmental factors. Urgent measures to preserve relict and endemic species are therefore necessary. The study of genetic diversity is a main element in the strategy for preserving natural populations of endemic species, as genetic diversity arises as a result of adaptations to certain environmental conditions and/or geographical isolation of populations [62,63,64]. Due to a lack of specialized molecular genetic markers, analyzing the biodiversity of rare and poorly studied endemic species is more difficult. Each system of molecular genetic markers has its own unique features, limitations, and area of effective application. Effective molecular approaches should cover the variability of the genome as a whole and correlate with the phenotypic variability of the species [12]. The study of the genetic biodiversity of the genus *Allium* is carried out using the analysis of individual genes from the chloroplast and nuclear genome [65,66]. The application of these approaches is effective for species identification. As a rule, comparative analysis of the internal transcribed spacer (ITS) of the ribosomal RNA gene cluster and gene sequences for the chloroplast genome are used for species identification [67]. The use of a PCR-based DNA profiling method, such as RAPD [18], ISSR [19], or AFLP [20], is ideal for molecular genetics and population studies for both eukaryotes and prokaryotes. A number of limitations associated with the specificity of each of these methods are associated with technical problems, such as sensitivity to DNA quality, PCR conditions, or high cost [68]. When developing SSR markers, NGS analysis is required for genetically remote genotypes for each species under study [69,70]. Using markers is preferable for rare or poorly studied species limited by a narrow distribution area, as they do not require large-scale and expensive primary work, unlike the development of SSR markers [65,71]. In this case, PCR-based DNA profiling is the best choice. Molecular genetic markers with a wide genome coverage, relatively even distribution, and universal use for any species, regardless of taxonomic affiliation, will be preferable to others studying genetic polymorphism. Also, these marker systems should be universal for use on different types of plants. For example, such markers can be supplemented with methods based on conservative interspersed repeat sequences in genomes, including a range of transposable elements, 5 S rRNA- and tRNA-related sequences, regions of certain promoters, and introns [44,62,63,64]. The most important criteria (prevalence in the genome and versatility of use at various sites) determine the choice of a molecular genetic approach for assessing the biodiversity of any plant species. Such markers are the most optimal for species with an unknown genome, or that are narrowly localized and/or vegetatively inactive. Based on the study of genetic polymorphism, the iPBS DNA profiling method is the most suitable of all the approaches for analyzing the population diversity of rare and poorly studied species. Moreover, such markers may be useful for assessing the mechanisms of adaptation to stressful environmental conditions because the activity of LTR retrotransposons causes a specific response of the organism to stress. Stress-induced activity of transposable elements can potentially affect the expression of neighboring genes. The revealed polymorphism using iPBS amplification DNA profiling for both individuals and a population within a species may indicate relatively recent events, i.e., the activity of transposable elements [72]. Under the stressful conditions of the Altai Mountains, which are characterized by significant daily temperature drops, high radiation levels, and summer snowfalls, we observed differences between populations of endemic *A. ledebourianum*, which manifested themselves both in the morphometric parameters of plants and on the genome level. The extreme nature of environmental factors has an extremely negative effect on the safety of young seedlings, resulting in *A. ledebourianum* populations with dissimilar spatial structures, from scattered to densely diffuse, depending on their conditions of existence. Population LD64, located at an altitude of 1920 m above sea level, under conditions of intensively developed deciduous litter that allows seeds and young seedlings to gain foothold, has the densest diffuse structure. This population is characterized by the presence of generative and vegetative individuals, and is a full member, which indicates relatively favorable environmental conditions. However, population LD6, located at a similar height, is characterized by a dispersed structure and a decrease in the number of generative individuals, and is aging. The absence of leaf litter and the movement of thawed waters determine the diffuse structures in the LD6 and LD20 populations. As a result, the soil horizon is eroded together with seeds that fell off in autumn and juveniles. Young plants do not have time to form a sufficiently developed root system and can also be washed away by water. Analysis of the morphometric parameters of *A. ledebourianum* plants in the studied populations made it possible to identify the ecological optimum for this species. The height of generative shoots in the flowering phase, the number of formed flowers, and seed production values have the highest values for populations inhabiting moderately humid meadows. Excessive moisture inherent in hummocky moraine significantly reduces the productivity of generative organs. Based on the results of the morphometric parameters of plants in the studied populations, we conclude that moderately wet meadows are optimal conditions for the existence of this species. The presence of young seedlings 1–2 years of age was revealed only in the LD20 and LD64 populations, which also confirms our assumption about the well-being of the ecological conditions of these populations. However, favorable conditions of humidification and illumination, contributing to the development of flora, also determine the direction of human economic activity. In areas of abundant grass, active work is observed to collect and harvest *A. ledebourianum*, as well as intensive grazing of domestic animals. Therefore, human economic activity is the main limiting factor of this type [11,73]. According to biodiversity studies of *A. ledebourianum*, more precisely about the study of the biological characteristics of the species during its introduction, or the results of biochemical studies are presented in the following works [51,72,74]. Studies of genetic polymorphism using genome in situ hybridization (GISH) and phylogenetic analysis have been carried out on this species of *Allium* [75,76,77]. Moreover, a significant part of the genetic diversity studies of *Allium* species have been carried out on cultivated species (*A. sativum*, *A. sepa*), or on species of breeding value [78,79,80,81,82]. In this work, we first tested the analysis of the molecular genetic diversity of natural populations of endemic *A. ledebourianum* using the DNA profiling method. Our previous studies of molecular genetic analysis of the biodiversity of the relict species *Allium altaicum* showed a high level of detectable polymorphism in the study of natural populations. The research results presented in this work are similar to the results of our previous studies on *A. altaicum*. Using the iPBS amplification profiling method, we observed the influence of the population’s altitude on the level of population variability [31]. *A. altaicum* populations located in favorable midland conditions showed a high level of genetic polymorphism, which was caused by the presence of several reproduction strategies, i.e., clonal and by seed germination. Also, we observed higher indicators of diversity for populations located in remote areas of Altai (Kalbinskiy Altai). In our studies, no significant dependence of the effect of height on the productivity of *A. ledebourianum* plants was revealed in the *A. ledebourianum* populations, or in the indicators of genetic diversity. Comparing the biodiversity indicators, we note that the values of the Shannon diversity index and the number of effective polymorphic amplicons were higher in the *A. ledebourianum* populations, but the values of heterozygosity were lower in *A. ledebourianum* populations (μHe = 0.136) compared to *Altai* onion populations (μHe = 0.145). Higher values of biodiversity indices in *A. ledebourianum* populations are possibly associated with the dominance of seed reproduction, which creates a higher level of diversity, in contrast to the clonal one, in which the daughter bulbs are genetically identical to the parent plant [82]. Certain studies show that the ability for vegetative reproduction, inherent in *Allium* species, is a peculiar way of ensuring the genetic stability of populations and preserving the genetic diversity of populations, and reducing losses from genetic erosion [83,84]. The results of our study revealed average levels of intrapopulation polymorphism in the samples of *A. ledebourianum* populations using the iPBS amplification profiling method. Estimating the biodiversity level of *A. ledebourianum* populations using iPBS amplification markers, we note the average level of genetic variability (since He < μHe for all populations) associated with inbreeding. This phenomenon is typical for endemic species with small populations, for which even a slight change in allele frequencies can have a significant impact on the biodiversity level, as the number of homozygous individuals increases [85]. The average level of genetic variability between *A. ledebourianum* populations may be due to their recent isolation, as well as to the absence of genetic drift that is caused by the ecological and biological characteristics of this species, which is narrowly adapted to places of increased moisture. The maximum adaptation to specific environmental conditions, acquired during the evolution of this species, is manifested by the presence of a short flowering period, which is possible even under snow cover, an abundant number of flowers on the shoot, and high potential seed production. All this allows them to maintain their genetic diversity in changing environmental conditions. Analysis of molecular variance showed that most of the molecular variability is due to interpopulation differences (67%), although individual populations (LD64 and LD6) living in favorable conditions show a relatively high level of intrapopulation polymorphism. Correlation analysis of quantitative characteristics, carried out using Spearman’s rank correlation coefficient, confirms our assumption that the existence of *A. ledebourianum* plants at different heights cannot be considered a factor determining the interpopulation diversity of this endemic species. Correlation analysis (Table S4) revealed that only iPBS amplicon polymorphism correlates with shoot height, plant productivity, and indicators of genetic variation in *A. ledebourianum* (Shannon diversity index and population heterozygosity). Our studies have shown that the most distant population (LD64) has the lowest allelic diversity (Na = 0.971), which is associated with geographic isolation. In the UPGMA cluster analysis based on the iPBS amplification profiling data, the populations were distributed according to their geographic location. We observe that the populations LD6 and LD20, located relatively close to each other, demonstrate genealogical closeness on a dendrogram, which indicates the average level of their differentiation. In the evolutionary past it was likely one large population, divided by the fragmentation of the area. In this work, for the first time, we analyzed the molecular genetic diversity of samples from natural populations of *A. ledebourianum*, which are endangered due to anthropological factors. *A. ledebourianum* is a vegetatively sedentary species, which, along with the spatial isolation of populations, is one of the reasons for its endemism. Despite the genetic diversity of each isolated *A. ledebourianum* population being closed and not supported from the outside, the genetic diversity in general is preserved within different populations. Therefore, each of these populations untouched by anthropogenic factors must be protected to maintain a certain level of genetic variability. Attention should also be paid to the preservation of the habitats of this endemic species, to exclude grazing, mowing, and other human economic activities. Due to its limited distribution, *A. ledebourianum* is highly vulnerable in terms of genetic biodiversity conservation, the study of which is very important against the high level of ecological degradation faced by the species.

## 5. Conclusions

In conclusion, this study presents a detailed analysis of narrowly endemic *A. ledebourianum* using the iPBS amplification profiling method and first reliable genetic data of its population structure. Both are essential for maintaining the genetic diversity of this endemic species. Therefore, high levels of intra- and interpopulation polymorphism were detected in natural populations of endemic *A. ledebourianum* using informative iPBS amplification markers, which was possible due to its simple, reproducible, and genome-wide distribution. Genetic diversity data, population structure, and genetic relationships between populations through iPBS amplification analysis will be helpful for *A. ledebourianum* germplasm management and for assisting conservation. For the first time, we present results of a biodiversity study using iPBS amplification profiling markers for the narrow endemic *A. ledebourianum* species. We investigated the genetic structure of populations that are important for the conservation of its genetic diversity. Our research improved understanding of the genetic diversity of natural populations of the relict *A. ledebourianum* species in the Altai territory. These findings may facilitate the development of a restoration strategy for the endangered species. Thus, highly informative iPBS amplification markers are effective in studying intra- and interpopulation polymorphisms of natural *A. ledebourianum* populations due to their simplicity, reproducibility, and wide distribution throughout the genome. This study revealed that the populations of *A. ledebourianum* are distinguished by an average level of genetic diversity, which is associated with adaptation to highly specific habitats. The iPBS amplification method may address the problem of species identification of the rare and endangered *A. ledebourianum* and the preservation of its biological diversity. The *A. ledebourianum* samples from the most distant populations can be used for the preservation and reproduction of the gene pool of this valuable plant species.

## Figures and Tables

**Table 1:** Sequence information of primer binding sites (PBS) primers used to assess genetic diversity of *A. ledebourianum* and their features in the present study. Sequences of PBS primers used in this study and comparative analysis of products of iPBS amplification of the DNA of *A. ledebourianum* populations.

Primer ID	Sequence 5′–3′	T_m_ (°C) *	Total Bands	Polymorphism Loci (%)	Polymorphism Information Content
2228	CATTGGCTCTTGATACCA	50.2	258	48.7	0.763
2240	AACCTGGCTCAGATGCCA	54.7	195	38.1	0.965
2395	TCCCCAGCGGAGTCGCCA	53.0	175	41.6	0.886
Mean			209	42.8	

* T_m_ (melting temperature), calculated with 1 μM concentration and without Mg^2+^ [58].

**Table 2:** Ecological and phytocenotic conditions of *A. ledebourianum* populations.

	Population		
	LD20	LD6	LD64
Geographical Coordinates	Western Altai. Northwestern foot of the Ivanovsky ridge, the Gray Lug tract, northwestern slope	Western Altai, southern part of Lineisky ridge, top of the Barsuk river	Southern Altai, Sarymsakty ridge, southeastern Foothills, Tautekeli river valley, park larch forest
	50°21′27″ N 83°53′54″ E 1170 m above sea level	50°19′12″ N 84°11′49″ E 1925 m above sea level	49°05′37″ N 86°12′36″ E 1925 m above sea level
Soil moistening	Moderate	Excessive	Moderate
Soil	Mountain-meadow	Wetland meadows	Mountain meadow
Subsoil	Moderately developed, 5–7 cm	Not developed, represented by litter	Intensively developed, more than 10 cm
Spatial structure of the population	Diffuse scattered	Diffused	Densely diffuse, spots of 3–5 individuals
Projective cover	75%	75%	90%
Phytocenosis	*Calamagrostis purpurea, Filipendula ulmaria, Veratrum lobelianum Bernh., Allium ledebourianum, Poa remota, Alopecurus pratensis, Veratrum lobelianum.*	*Filipendula ulmaria *(L.)* Maxim.*, *Phalaroides arundinaceae *(L.)* Rauschert*, *Calamagrostis purpurea (Trin.) Trin.*, *Allium ledebourianum. Lathyrus pratensis* L., *Cerastium davuricum Fisch. ex Spreng.*, *Carex rostrata Stokes*, *Alopecurus pratensis* L., *Poa sibirica Roshev.*, *P. remota Forsell*.	*Calamagrostis epigeios* (L.) Roth, *Elymus mutabilis* (Drob.) Tzvel.

**Table 3:** Characteristics of *A. ledebourianum* plants from natural populations in Kazakhstan Altai.

Signs	LD20	LD6	LD64
Shoot height during the flowering phase (min/max), cm	93/108	32/53	65/84
Average height, cm	99.6 ± 1.2	44.8 ± 2.9	68.2 ± 3.4
Number of inflorescences per bush	6	5	4
Number of flowers per inflorescence	80.8	69.1	87.2
Number of formed bolls per inflorescence	33.9	36.6	64.6
Fruit formation coefficient, %	41.9	52.9	74.1
Number of bolls per bush	995.0	647.0	565.0
Boll formations per bush, %	98.0	95.0	93.0
Potential seed production of one generative shoot, pcs.	484.8	414.6	522.0
Actual seed production of one generative shoot, pcs.	140.1	91.5	161.5
Productivity rate, %	28.9	22.1	30.9
Number of seeds per inflorescence	1194.0	725.0	678.0
Weight 1000 pcs, g	1.31	1.22	1.11
Seed length, mm	3.46 ± 0.25	3.08 ± 0.31	3.19 ± 0.24
Seed width, mm	2.01 ± 0.25	2.32 ± 0.24	1.98 ± 0.55
Ground germination, %	28	48	61.2
Laboratory germination, %	73	71.5	83.2

## Data Availability

Data is contained within the article or supplementary material.

## Supplementary Materials

The following are available online at https://www.mdpi.com/article/10.3390/biotech10040023/s1, Table S1: Statistical measure of genetic diversity of *A. ledebourianum*, based on inter-primer binding-site markers; Table S2: Summary AMOVA analysis of *A. ledebourianum* populations using iPBS markers; Table S3: Summary statistics of Spearman’s rank for morphological and vegetation traits of *A. ledebourianum* in wild populations; Table S4: Summary statistics of Spearman’s rank correlations for shoot height, fruit formation coefficient, productivity rate, and soil germination of *A. ledebourianum* with the genetic variation.
