# Supplementary material for: Primer Binding Site (PBS) Profiling of Genetic Diversity of Natural Populations of Endemic Species Allium ledebourianum Schult
Source: BioTech (Basel). 2021 Oct 13;10(4):23. doi: 10.3390/biotech10040023 (PMC9245474; doi:10.3390/biotech10040023)
Supplement: Supplementary file 1 [file biotech-10-00023-s001.zip › biotech-1311945-supplementary.pdf]

Supplementary Materials

Primer Binding Site (PBS) Profiling of Genetic Diversity of Natural Populations of Endemic Species *Allium ledebourianum* Schult.

Oxana Khapilina [1,]*, Ainur Turzhanova [1], Alevtina Danilova [2], Asem Tumenbayeva [1], Vladislav Shevtsov [1], Yuri Kotukhov [2], Ruslan Kalendar [3,4,]*

[1] National Center for Biotechnology, Korgalzhin hwy 13/5, 010000, Nur-Sultan, Kazakhstan; turzhanova-ainur@mail.ru (A.T.); asem.tumenbaeva2016@mail.ru (A.T.); xatabadich@gmail.com (V.S.)
[2] Altai Botanical Garden, Yermakova Str 1, 070000, Ridder, Kazakhstan; a-n-danilova@yandex.ru (A.D.); sumbembayev@gmail.com (Y.K.)
[3] National Laboratory Astana, Nazarbayev University, 010000, Nur-Sultan, Kazakhstan
[4] Helsinki Institute of Life Science HiLIFE, Biocenter 3, Viikinkaari 1, University of Helsinki, FI-00014, Helsinki, Finland

* **Correspondence:** oksfur@mail.ru (O.K.); ruslan.kalendar@helsinki.fi (R.K.); Tel.: +358294158869 (R.K.)

**O.K.**: oksfur@mail.ru	ORCID 0000-0002-7256-568X ScopusID 57194829297
**A.T.**: turzhanova-ainur@mail.ru	Scopus 57216895766
**A.D.**: a-n-danilova@yandex.ru	Scopus 57221397308
**A.T.**: asem.tumenbaeva2016@mail.ru	Scopus 57216894029
**V.S.**: xatabadich@gmail.com	Scopus 57216896596
**Y.K.**: sumbembayev@gmail.com	ORCID 0000-0002-7700-5390 ScopusID 56946858600
**R.K.**: ruslan.kalendar@helsinki.fi	ORCID 0000-0003-3986-2460 ScopusID 6602789279

Running Head: DNA profiling and assessment of genetic diversity of endemic *Allium ledebourianum*

Abbreviations: PBS: primer binding site; LTR: long terminal repeat

Key words: *Allium ledebourianum* Schult.; DNA molecular marker; genetic diversity; iPBS amplification

Supplementary Table S1. Statistical measure of genetic diversity of *A. ledebourianum*. based on inter-primer binding-site markers.

Population ID	N	NTI	PPL (%)	I	He	uHe	Na	Ne
LD6	11	251	34.3	0.198	0.131	0.137	1.257	1.218
LD20	11	221	40.0	0.165	0.108	0.114	1.114	1.178
LD64	11	156	40.0	0.223	0.152	0.159	0.971	1.270
MV			38.1	0.195	0.130	0.136	1.114	1.222
SE			1.9%	0.027	0.018	0.019	0.078	0.033

Supplementary Table S2. Summary AMOVA analysis of *A. ledebourianum* populations using iPBS markers.

Source	df	SS	MS	Est. Var.	%	ϕPT
Among Pops	2	120.909	60.455	5.264	67	
Within Pops	30	76.545	2.552	2.552	33	0.674
Total	32	197.455		7.815	100	

Levels of significance are based on 999 iteration steps and are indicated by three ($p<0.001$) asterisks SS the sum of squares. MS mean squares. % proportion of genetic variability

Supplementary Table S3. Summary statistics of Spearman's rank for morphological and vegetation traits of *A. ledebourianum* in wild populations.

**Sign**	**Ground germination (%)**	**Weight 1000 pcs. g**	**Number of seeds per inflorescence**	**Semenification rate (%)**	**Real seed production of one generative shoot. pcs.**	**Potential seed production of one generative shoot. pcs.**	**% of boll formation per bush**	**Number of bolls per bush**	**Fruit formation (%)**	**Number of bolls per inflorescence. pcs.**	**Number of flowers per inflorescence. pcs.**	**Shoot height in the flowering phase (min/ max). cm**
Height above sea level. m	0.919	-0.836	-0.997	-0.300	-0.219	-0.175	-0.918	-0.984	0.762	0.567	-0.167	-0.827
Shoot height in the flowering phase (min/max). cm	-0.538	-0.383	0.778	0.785	0.730	0.698	0.536	0.713	-0.266	-0.006	0.692	
Number of flowers per inflorescence. pcs.	0.236	-0.402	0.085	0.991	0.999*	1.000**	-0.239	-0.013	0.512	0.718		
Number of bolls per inflorescence. pcs.	0.846	-0.926	-0.633	0.615	0.679	0.712	-0.848	-0.706	0.966			
Fruit formation (%)	0.956	-0.992	-0.813	0.389	0.465	0.505	-0.957	-0.866				
Number of balls per bush	-0.975	0.921	0.995	0.124	0.040	-0.005	0.974					
% of boll formation per bush	-1.000**	0.985	0.947	-0.103	-0.187	-0.231						
Potential seed production of one generative shoot. pcs.	0.228	-0.395	0.093	0.992	0.999*							
Real seed production of one generative shoot. pcs.	0.184	-0.353	0.138	0.996								
Productivity rate.%	0.101	-0.273	0.221									
Number of seeds per inflorescence	-0.948	0.878										
Weight 1000 pcs. g	-0.985											

Significance values are ** = p≤0.001; * = p≤0.05 The F-values are provided with significance level indicated by the asterisks

Supplementary Table S4. Summary statistics of Spearman´s rank correlations for shoot height. fruit formation coefficient. productivity rate and soil germination of *A. ledebourianum* with the genetic variation.

**Variables**	**ϱ**	**p**	**Sign**
Shoot height			
SI	0.63	0.56	n.s.
PB	0.9	0.000	***
He	0.58	0.000	n.s
Fruit formation coefficient			
SI	0.59	0.6	n.s.
PB	0.18	0.000	***
He	0.63	0.56	n.s.
Productivity rate			
SI	0.97	0.14	n.s.
PB	0.98	0.000	***
He	0.96	0.17	n.s.
Soil germination			
SI	0.32	0.79	n.s.
PB	-0.12	0.000	***
He	0.37	0.75	n.s.
